# Genome-Wide Characterization of CAPE-Producing *PR1* Genes Reveals Regulator-Dependent Expression and Abiotic Stress-Associated Functions in *Nicotiana tabacum*

**DOI:** 10.3390/plants15121801

**Published:** 2026-06-11

**Authors:** Yuanxin Wu, Zhongqi Zhang, Oluwaseyi Setonji Hunpatin, Zhongyang Liu, Shamima Naznin, Tao Liu, Jie Wang, Songxiao Cao, Zenglin Zhang, Yongfeng Guo, Sayed Abdul Akher, Zhenbiao Zhang

**Affiliations:** 1 Tobacco Research Institute, Chinese Academy of Agricultural Sciences, Qingdao 266101, China; 15703782950@163.com (Y.W.); seyipatin@gmail.com (O.S.H.); liutao131401@163.com (T.L.); wangjie06@caas.cn (J.W.); zhangzenglin@caas.cn (Z.Z.); guoyongfeng@caas.cn (Y.G.); 2 Graduate School of Chinese Academy of Agricultural Sciences, Beijing 100081, China; 3 Heze Academy of Agricultural Sciences, Heze 274000, China; soybean2021@163.com (Z.Z.); liukezhang1990@163.com (Z.L.); 4 Department of Plant Pathology, Hajee Mohammad Danesh Science and Technology University, Dinajpur 5200, Bangladesh; shamima.naz02@gmail.com; 5 Donggang District Bureau of Agriculture and Rural Affairs, Rizhao 276800, China; hanhaic@126.com

**Keywords:** CAPE peptides, *Nicotiana tabacum*, abiotic stress, Solanaceae

## Abstract

Pathogenesis-related 1 (PR1) proteins are important components of plant defense and stress responses and also serve as precursors of CAP-derived peptides (CAPE), a class of small bioactive peptides involved in immune and stress signaling. Despite their potential biological significance, CAPE-producing *PR1* genes have not been systematically characterized in tobacco (*Nicotiana tabacum*). In this study, a genome-wide analysis identified 17 CAPE-producing PR1 genes, designated *NtCAPE1* to *NtCAPE17*, in the tobacco genome. These genes encode proteins containing conserved CAP domains and N-terminal signal peptides, with predicted hydrophilic properties and mainly vacuolar localization, indicating conserved structural features within the family. Phylogenetic analysis, gene structure organization, conserved motif profiling, chromosomal distribution, and synteny analyses revealed both evolutionary conservation and duplication-driven diversification of the *NtCAPE* family. Promoter cis-element analysis showed enrichment of regulatory elements associated with phytohormone signaling, development, and stress responses. Public transcriptomic datasets revealed dynamic and gene-specific expression patterns under water-deficit and salinity stress, and qRT-PCR analysis further confirmed the stress-responsive expression of selected *NtCAPE* genes. Functional assays using synthetic mature peptides showed that NtCAPE9 and NtCAPE17 alleviated salinity stress- and osmotic stress-induced leaf yellowing, improved chlorophyll retention, suppressed senescence-associated responses, reduced H_2_O_2_ accumulation and POD activity, modulated stress-responsive gene expression, and promoted seed germination under salinity and osmotic stress, respectively. These results provide a comprehensive characterization of CAPE-producing *PR1* genes in tobacco and identify NtCAPE9 and NtCAPE17 as candidate stress-associated peptides with exogenous activity under salinity and osmotic stress conditions.

## 1. Introduction

The Solanaceae family comprises several agriculturally important crops, including tomato (*Solanum lycopersicum*), potato (*Solanum tuberosum*), pepper (*Capsicum annuum*), eggplant (*Solanum melongena*), and tobacco (*Nicotiana tabacum*). However, the productivity and quality of these crops are increasingly constrained by abiotic stresses such as water-deficit stress, salinity, high temperature, and heavy metal toxicity, which disrupt physiological homeostasis and reduce yield [1]. To cope with these adverse conditions, plants have evolved complex adaptive mechanisms involving coordinated hormone signaling, transcriptional reprogramming, and activation of defense-related proteins [2,3,4].

Among these proteins, Pathogenesis-Related 1 (PR1) members are widely recognized as hallmarks of salicylic acid (SA)-mediated defense and systemic acquired resistance [5]. Beyond serving as molecular markers, PR1 proteins belong to the CAP superfamily and have been shown to perform active biological functions [5,6,7]. A notable advance in this field was the discovery that certain PR1 proteins harbor short bioactive peptides, termed CAP-derived peptides (CAPE), embedded within their conserved C-terminal region [5,8]. Proteolytic processing releases these peptides, enabling them to function as endogenous immune regulators [9,10]. In tomato, CAPE1 derived from PR1 was identified as a wound-induced peptide capable of activating defense responses [11]. In Arabidopsis, cleavage of PR1 by the cysteine protease XCP1 generates CAPE peptides required for full systemic immunity, linking regulated PR1 processing to long-distance defense signaling [9]. These findings shift the view of PR1 proteins from passive defense markers to precursors of functional peptide signals.

Genome-wide surveys in several crop species have revealed that *PR1* gene families are evolutionarily conserved yet variable in size due to lineage-specific duplication events [12,13,14,15,16]. Expression analyses consistently demonstrate that many *PR1* genes are inducible by biotic and abiotic stresses and enriched in hormone- and stress-responsive *cis*-elements [17,18]. Despite the emerging recognition of PR1-derived CAPE peptides as bioactive signals, their repertoire, evolutionary diversification, and stress-associated functions remain largely unexplored in tobacco. Here, we performed a genome-wide analysis of CAPE-producing *PR1* genes in *N. tabacum* and integrated phylogenetic, structural, syntenic, promoter, and expression analyses to define the evolutionary and regulatory features of this gene family. By combining public transcriptomic datasets with qRT-PCR validation, we further identified NtCAPE members that respond dynamically to water-deficit stress and salinity stress. Finally, functional assays using synthetic mature peptides revealed that *NtCAPE9* and *NtCAPE17* mitigate salinity- and water-deficit stress-induced damage, respectively. These findings establish a comprehensive framework for the NtCAPE family in tobacco and highlight selected CAPE peptides as candidate endogenous regulators of abiotic stress adaptation.

## 2. Results

### 2.1. Genome-Wide Identification and Characterization of NtCAPE Genes

Using genome-wide identification based on conserved CAPE-producing PR1 domains, a total of 17 CAPE-producing *PR1* genes were identified in *N. tabacum* and designated as *NtCAPE1-NtCAPE17* according to their chromosomal positions (Table 1). All 17 NtCAPE proteins possess the conserved CAP domain and a C-terminal region matching the CAPE peptide signature (PPGNxxxxPY), confirming their identity as bona fide CAPE-producing PR1 precursors. Physicochemical characterization revealed that the predicted NtCAPE proteins ranged from 161 to 201 amino acids (AA) in length, with NtCAPE3 and NtCAPE6 representing the shortest proteins (161 aa), while NtCAPE9 was the longest (201 aa). The predicted molecular weights varied from 17.84 kDa (NtCAPE1) to 23.32 kDa (NtCAPE9), which is consistent with the typical size range of PR1 family proteins. The theoretical isoelectric point (pI) ranged from 4.83 to 8.92, indicating that most NtCAPE proteins are weakly acidic to neutral, while a few members exhibit slightly basic characteristics, suggesting moderate variation in protein charge within the family. Instability index analysis showed values ranging from 16.66 (NtCAPE1) to 44.52 (NtCAPE2), indicating that the majority of NtCAPE proteins are predicted to be stable under physiological conditions, whereas only a few members may exhibit relatively higher structural instability. The aliphatic index ranged from 57.32 (NtCAPE2) to 77.52 (NtCAPE3), suggesting moderate thermostability among NtCAPE proteins. The grand average of hydropathicity (GRAVY) values ranged from −0.516 (NtCAPE5) to −0.116 (NtCAPE1), and all NtCAPE proteins exhibited negative GRAVY scores, indicating hydrophilic properties typical of secreted proteins. Consistently, signal peptide prediction revealed that all NtCAPE members contain N-terminal signal peptides, supporting their targeting through the classical secretory pathway. Subcellular localization prediction further indicated that all NtCAPE proteins are localized in the vacuole (17 members, 100%), suggesting a conserved intracellular localization pattern within the family. The enrichment of NtCAPE proteins in the vacuole implies their potential involvement in intracellular protein processing, storage, or stress-related peptide release associated with CAPE peptide maturation. Overall, the identification and basic characterization analysis demonstrate that NtCAPE proteins are structurally conserved but exhibit moderate physicochemical variation, providing a molecular basis for the functional diversification of CAPE-producing PR1 proteins in tobacco.

### 2.2. Phylogenetic Relationships, Gene Structure, and Conserved Motifs of NtCAPE Genes

**Figure 1:**
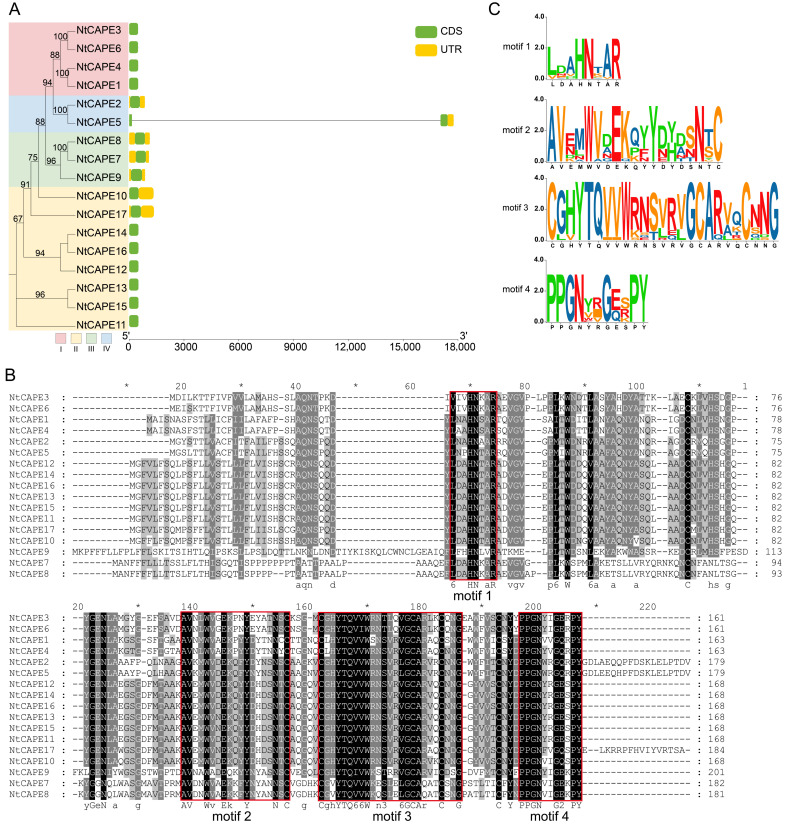
Phylogenetic relationships, gene structure, and conserved motif analysis of *NtCAPE* genes. (**A**) Phylogenetic tree and gene structure organization of *NtCAPE* genes. NtCAPE proteins were classified into four clades (I–IV) based on sequence similarity. Gene structures are shown with coding sequences (CDS, (**B**)) Multiple sequence alignment of NtCAPE proteins showing conserved regions across the CAP domain. Conserved motifs identified by MEME analysis are indicated by red boxes. Asterisks indicate fully conserved amino acid residues among the aligned NtCAPE protein sequences. (**C**) Sequence logos of the four conserved motifs (motifs 1–4) identified in NtCAPE proteins, showing amino acid conservation patterns within each motif. Motif 4 corresponds to the conserved C-terminal CAPE peptide region containing the characteristic PPGNxxxxPY signature.

To investigate the evolutionary relationships and structural characteristics of *NtCAPE* genes, protein sequence-based phylogenetic analysis was integrated with annotation-based gene structure comparison from the *N. tabacum* genome, with the latter focused primarily on coding-region organization rather than on inferring regulatory variation from UTR length (Figure 1). Based on phylogenetic relationships, NtCAPE proteins were divided into four clades (I–IV), and genes within the same clade generally displayed similar structural features (Figure 1A). Clade I (*NtCAPE1*, *NtCAPE3*, *NtCAPE4*, and *NtCAPE6*) showed compact gene structures mainly composed of coding sequence (CDS) regions with little or no detectable UTRs. Clade II contained *NtCAPE2* and *NtCAPE5*, which exhibited relatively greater structural variation; notably, *NtCAPE5* displayed a markedly extended genomic span compared with other family members. Clade III (*NtCAPE*7–*NtCAPE*9) showed moderate structural variation, with several genes containing UTR regions on both sides of the CDS. In contrast, most members of Clade IV (*NtCAPE10–NtCAPE17*) exhibited highly conserved CDS-dominated structures, although *NtCAPE10* and *NtCAPE17* possessed relatively longer UTR regions. Overall, *NtCAPE* genes displayed simple and conserved gene structures across the family.

Multiple sequence alignment revealed high sequence conservation among NtCAPE proteins, particularly within the CAP domain (Figure 1B). MEME analysis identified four conserved motifs (motifs 1–4) that were widely distributed among *NtCAPE* members (Figure 1C). Motif 1 was mainly located in the N-terminal region, whereas motif 2 occurred in the central region and showed moderate variability. Motif 3 corresponded to the highly conserved CAP domain core and was present in all NtCAPE proteins, indicating its essential structural role. Motif 4 was located at the C-terminus and represented the conserved CAPE peptide region containing the characteristic PPGNxxxxPY signature. Sequence logo analysis further confirmed strong conservation of key residues within each motif, while minor variation in surrounding amino acids suggests potential functional diversification among NtCAPE proteins. Together, these analyses indicate that *NtCAPE* genes are evolutionarily conserved and structurally similar, while subtle variations in gene organization and motif composition may contribute to functional differentiation within the tobacco CAPE-producing *PR1* family.

### 2.3. Chromosomal Distribution, Synteny Analysis, and Pairwise Sequence Similarity of NtCAPE Genes

**Figure 2:**
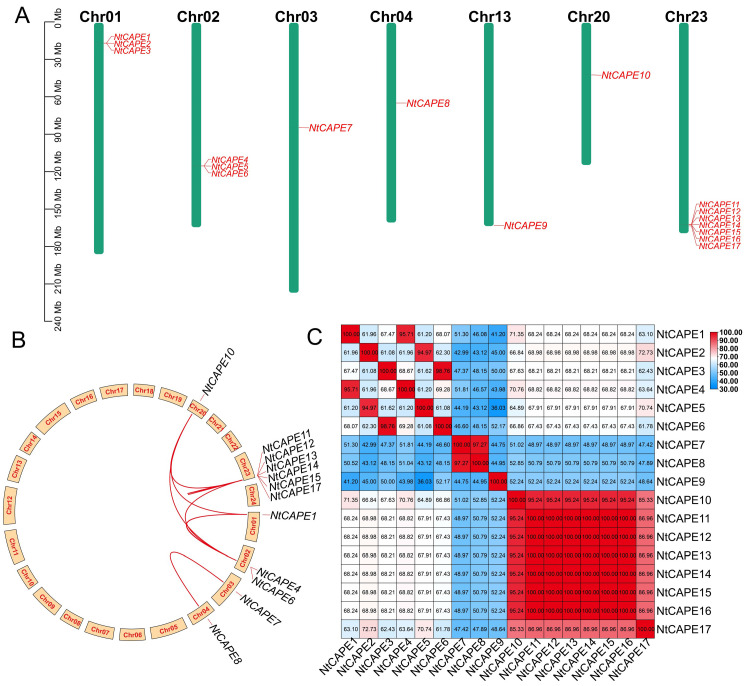
Chromosomal distribution, intra-genomic synteny, and pairwise sequence similarity analysis of *NtCAPE* genes in *N. tabacum*. (**A**) Chromosomal locations of 17 *NtCAPE* genes across seven tobacco chromosomes. (**B**) Intra-genomic synteny analysis showing collinear relationships among *NtCAPE* genes; red lines indicate duplicated gene pairs. (**C**) Pairwise sequence similarity heatmap of NtCAPE proteins based on full-length amino acid sequences, showing high conservation among several members, especially *NtCAPE11–NtCAPE16*.

Chromosomal mapping showed that the 17 *NtCAPE* genes are unevenly distributed across seven chromosomes of *N. tabacum*, including Chr01, Chr02, Chr03, Chr04, Chr13, Chr20, and Chr23 (Figure 2A). Three genes were located on Chr01 (*NtCAPE1–NtCAPE3*) and three on Chr02 (*NtCAPE4–NtCAPE6*), forming local gene clusters. Single genes were present on Chr03 (*NtCAPE7*), Chr04 (*NtCAPE8*), Chr13 (*NtCAPE9*), and Chr20 (*NtCAPE10*). Notably, Chr23 contained the largest cluster comprising seven genes (*NtCAPE11–NtCAPE17*). Based on genomic proximity (within 200 kb) and MCScanX analysis, the Chr23 cluster was classified as a local gene cluster formed primarily through tandem duplication events, with subsequent segmental duplication contributing to the dispersal of some members to other chromosomes.

Intra-genomic synteny analysis revealed several collinear relationships among *NtCAPE* genes, indicating that segmental duplication contributed to gene family expansion (Figure 2B). Clear syntenic connections were observed between the Chr23 gene cluster and specific genes located on Chr01 (*NtCAPE1*), Chr02 (*NtCAPE4* and *NtCAPE6*), and Chr20 (*NtCAPE10*), suggesting that only a subset of genes on Chr01 and Chr02 participated in duplication events. In addition, genes located on Chr03 (*NtCAPE7*) and Chr04 (*NtCAPE8*) showed collinear relationships with each other. In contrast, *NtCAPE9* on Chr13 did not show obvious syntenic connections in this analysis, indicating that not all *NtCAPE* members were involved in large-scale duplication events. The non-synonymous substitution rate (Ka)/synonymous substitution rate (Ks) analysis further showed that all calculable whole-genome duplication (WGD)/segmental duplicated pairs had Ka/Ks values below 1, ranging from 0.089 to 0.278 (Supplementary Table S1). The tandem duplicated pairs *NtCAPE12/NtCAPE14* and *NtCAPE13/NtCAPE15* showed 0 value of Ka and Ks, indicating nearly identical coding sequences and therefore were not suitable for Ka/Ks-based selection inference (Supplementary Table S1).

To further assess evolutionary conservation among NtCAPE proteins, pairwise sequence similarity analysis was conducted using full-length amino acid sequences (Figure 2C). NtCAPE proteins exhibited moderate to high sequence similarity, indicating overall conservation within the CAPE-producing *PR1* family in *N. tabacum*. Notably, *NtCAPE11–NtCAPE16* displayed nearly identical sequences, suggesting possible recent duplication events and potentially similar biological functions. Similarly, *NtCAPE3* and *NtCAPE6*, as well as *NtCAPE7* and *NtCAPE8*, showed high similarity, indicating possible functional redundancy. In contrast, lower similarity values were observed between members from different phylogenetic groups, reflecting evolutionary divergence within the NtCAPE family. Overall, the combined analyses indicate that chromosomal clustering together with segmental duplication events, particularly involving the Chr23 gene cluster, may have contributed to the expansion and diversification of *NtCAPE* genes in tobacco.

### 2.4. Phylogenetic Analysis of CAPE-Producing PR1 Proteins Across Different Plant Species

**Figure 3:**
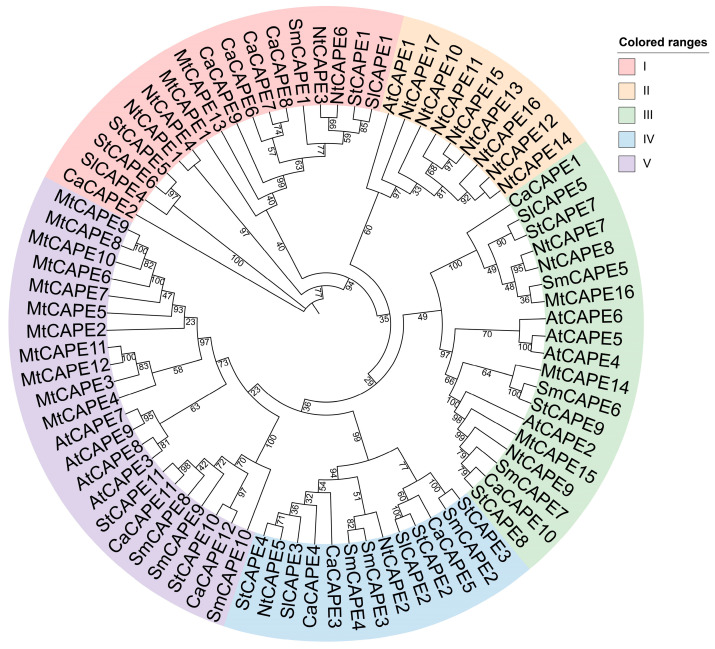
Phylogenetic analysis of CAPE-producing PR1 proteins from representative plant species. Circular phylogenetic tree constructed using full-length CAPE protein sequences from *N. tabacum* (*NtCAPE*), *A. thaliana* (*AtCAPE*), *S. lycopersicum* (*SlCAPE*), *S. tuberosum* (*StCAPE*), *C. annuum* (*CaCAPE*), *S. melongena* (*SmCAPE*), and *M. truncatula* (*MtCAPE*). CAPE proteins were classified into five subfamilies (I–V), indicated by different colored sectors. Numbers at the branch nodes represent bootstrap support values (%).

To investigate the evolutionary relationships of CAPE-producing PR1 proteins, a phylogenetic tree was constructed using CAPE proteins from *Arabidopsis thaliana (AtCAPE)*, *N. tabacum (NtCAPE)*, *S. lycopersicum (SlCAPE)*, *S. tuberosum (StCAPE)*, *C. annuum (CaCAPE)*, *S. melongena (SmCAPE)*, and *Medicago truncatula (MtCAPE)* (Figure 3). Based on phylogenetic relationships, CAPE proteins were classified into five distinct subfamilies (I–V), indicating both conservation and lineage-specific diversification.

Subfamily I contained CAPE proteins mainly from Solanaceae species and M. truncatula, and notably did not include *Arabidopsis* members. Subfamily II comprised *NtCAPE10–NtCAPE17* together with a single *Arabidopsis* member (AtCAPE1), suggesting possible lineage-biased diversification within this clade. Subfamily III was predominantly composed of Solanaceae CAPE proteins and included several NtCAPE members (*NtCAPE7–NtCAPE9*), together with *Arabidopsis* proteins *AtCAPE2* and *AtCAPE4–AtCAPE6*, indicating partial conservation between tobacco and *Arabidopsis* lineages. Subfamily IV consisted of CAPE proteins from different Solanaceae species but lacked representatives from *Arabidopsis* and Medicago, and contained only two tobacco members (*NtCAPE2* and *NtCAPE5*). In contrast, Subfamily V was dominated by CAPE proteins from *M. truncatula* and *Arabidopsis* and showed no representation from tobacco, suggesting possible lineage-biased diversification outside the Solanaceae group. Overall, the phylogenetic analysis demonstrates that NtCAPE proteins are distributed across multiple evolutionary clades, reflecting both conservation within Solanaceae species and diversification among different plant lineages.

### 2.5. Comparative Synteny Analysis of NtCAPE Genes Across Plant Species

**Figure 4:**
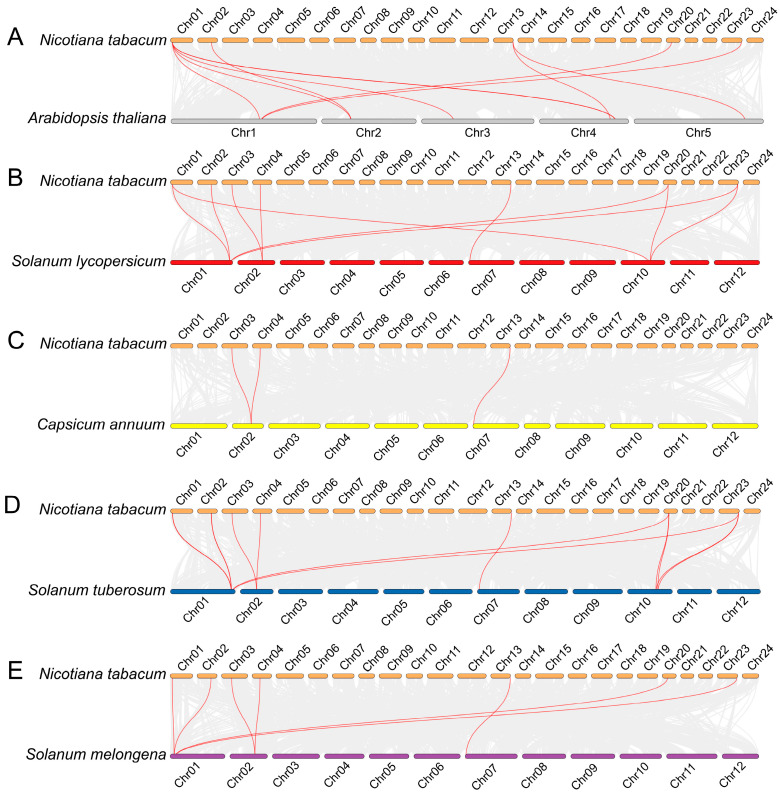
Comparative synteny analysis of *NtCAPE* genes across different plant species. (**A**–**E**) Syntenic relationships between *N. tabacum* and *A. thaliana* (**A**), *S. lycopersicum* (**B**), *C. annuum* (**C**), *S. tuberosum* (**D**), and *S. melongena* (**E**). Red lines indicate collinear gene pairs. Gray lines indicate background collinear blocks between genomes, while red lines highlight collinear gene pairs involving *NtCAPE* genes and their corresponding CAPE-producing *PR1* genes in the compared species. Detailed collinear gene pairs are listed in Supplementary Table S2.

To further investigate the evolutionary conservation of *NtCAPE* genes, comparative synteny analysis was performed between *N. tabacum* and five representative plant species, including *Arabidopsis thaliana*, *S. lycopersicum*, *C. annuum*, *S. tuberosum*, and *S. melongena* (Figure 4A–E), and the detailed collinear gene pairs are listed in Supplementary Table S2. The results revealed extensive collinear relationships between tobacco and the analyzed species. A total of 6, 8, 3, 9, and 7 *NtCAPE* genes showed syntenic relationships with CAPE-producing *PR1* genes in *A. thaliana*, *S. lycopersicum*, *C. annuum*, *S. tuberosum*, and *S. melongena*, respectively, corresponding to 10, 10, 3, 13, and 7 collinear gene pairs (Figure 4A–E; Supplementary Table S2). The number of detected collinear gene pairs varied among species, with *S. tuberosum* showing the highest number of pairs, followed by *A. thaliana* and *S. lycopersicum*, while fewer pairs were detected in *C. annuum* and *S. melongena*. Several *NtCAPE* genes exhibited conserved collinearity across multiple species, indicating evolutionary conservation of these genomic loci. Among them, *NtCAPE9* displayed syntenic relationships in all analyzed species, suggesting that it may represent an evolutionarily conserved member of the NtCAPE family. Overall, these findings suggest that both tandem and segmental duplication events contributed to the expansion of the *NtCAPE* gene family in tobacco, while interspecies synteny analysis highlights the balance between evolutionary conservation and lineage-specific diversification of CAPE-producing *PR1* genes during plant evolution.

### 2.6. Cis-Acting Regulatory Elements in NtCAPE Gene Promoters

**Figure 5:**
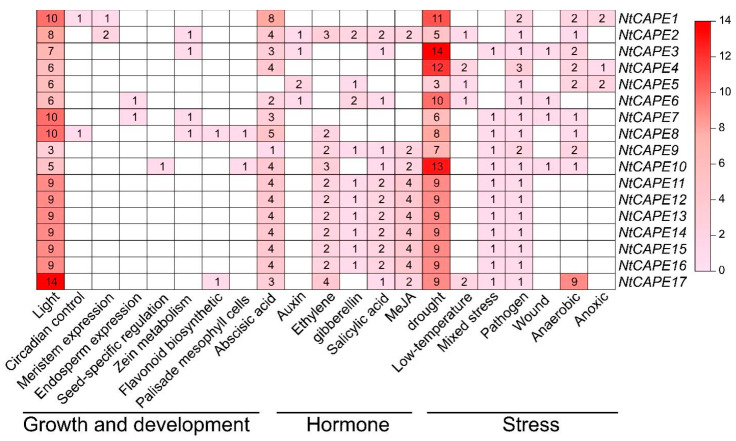
*Cis*-acting regulatory elements in the promoter regions of *NtCAPE* genes. Distribution and abundance of *cis*-acting elements related to growth and development, hormone responsiveness, and stress responses in *NtCAPE* promoters. Color intensity indicates the number of corresponding cis elements identified in each promoter.

Promoter analysis revealed that *NtCAPE* genes harbor abundant *cis*-acting elements associated with development, hormone signaling, and stress responses (Figure 5). Among these, light-responsive elements were the most abundant and widely distributed, suggesting that light is a major regulatory factor controlling *NtCAPE* gene expression. Hormone-responsive elements, particularly those related to abscisic acid and methyl jasmonate, were widely enriched, whereas auxin-, ethylene-, gibberellin-, and salicylic acid-responsive elements occurred at moderate levels. Stress-related elements were also highly represented, with water-deficit stress-responsive motifs showing strong enrichment across multiple NtCAPE promoters, indicating a potential role in water-deficit stress adaptation. These results indicate that *NtCAPE* gene expression is regulated by integrated developmental, hormonal, and environmental signals.

### 2.7. Descriptive Expression Profiling of NtCAPE Genes Under Water-Deficit Stress and Salinity Stress Based on Public RNA-Seq Data

**Figure 6:**
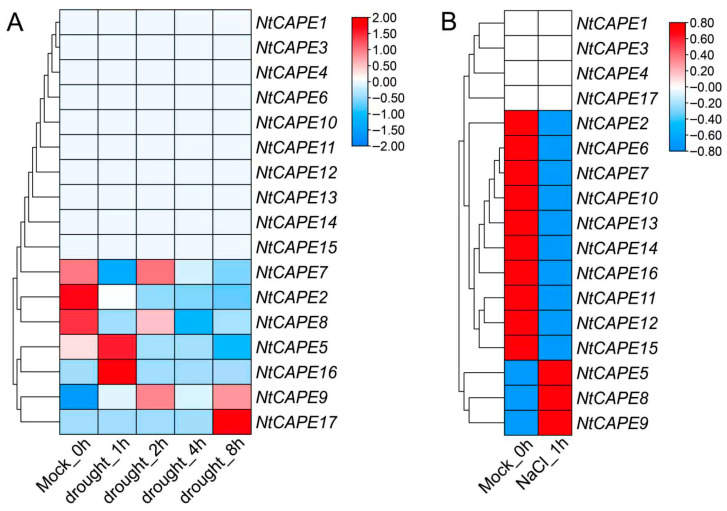
Expression profiles of *NtCAPE* genes under water-deficit stress and salinity stress based on public RNA-seq data. Heatmap showing the transcriptional responses of *NtCAPE* genes under water-deficit stress and salinity (NaCl) stress conditions using publicly available RNA-seq datasets. (**A**) Expression patterns of *NtCAPE* genes under water-deficit stress at different time points. (**B**) Expression patterns of *NtCAPE* genes under salinity (NaCl) stress. Gene expression levels are shown as normalized values and visualized using a color scale, with red indicating higher relative expression and blue indicating lower relative expression. Hierarchical clustering was performed to group genes with similar apparent expression patterns. Because this analysis was based on a heatmap visualization of normalized expression values, these data were interpreted as descriptive expression profiles rather than statistically defined differential expression.

Public RNA-seq datasets were used to obtain an overview of *NtCAPE* expression patterns under water-deficit stress and salinity stress conditions (Figure 6). Under water-deficit stress, most *NtCAPE* genes, including *NtCAPE1*, *NtCAPE3*, *NtCAPE4*, *NtCAPE6*, and *NtCAPE10–NtCAPE15*, showed relatively limited changes across the examined time points. In contrast, several members displayed apparent time-dependent variation. For example, *NtCAPE5* and *NtCAPE16* showed higher relative expression at 1 h, *NtCAPE9* showed increased relative expression at selected time points, particularly 2 h and 8 h, and *NtCAPE17* displayed the most prominent increase at 8 h. Notably, *NtCAPE2* and *NtCAPE8* showed relatively high expression in the Mock_0h sample but did not exhibit a clear water-deficit stress-induced pattern across subsequent time points (Figure 6A). Under salinity stress, *NtCAPE5*, *NtCAPE8*, and *NtCAPE9* showed higher relative expression in the NaCl_1h sample compared with Mock_0h, whereas several other genes, including *NtCAPE2*, *NtCAPE6*, *NtCAPE7*, *NtCAPE10–NtCAPE16*, showed lower relative expression. *NtCAPE1*, *NtCAPE3*, *NtCAPE4*, and *NtCAPE17* displayed relatively minor changes (Figure 6B). Together, these descriptive expression profiles suggested that *NtCAPE9* and *NtCAPE17* were suitable candidates for further validation, with *NtCAPE9* associated with the salinity-stress expression pattern and *NtCAPE17* showing a prominent late response under water-deficit stress/osmotic stress.

### 2.8. qRT-PCR Validation of NtCAPE Gene Expression Under Water-Deficit Stress and Salinity Stress

**Figure 7:**
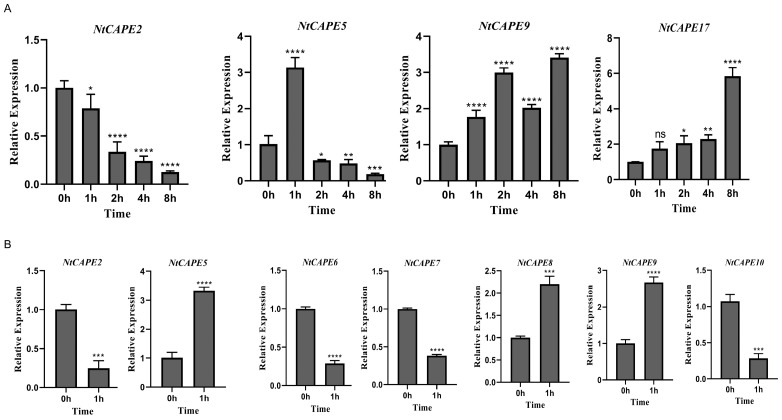
qRT-PCR validation of *NtCAPE* gene expression under water-deficit stress and salinity stress. Quantitative real-time PCR (qRT-PCR) analysis validating the expression patterns of selected *NtCAPE* genes under water-deficit stress and salinity (NaCl) stress conditions. (**A**) Relative expression levels of *NtCAPE* genes under water-deficit stress. (**B**) Relative expression levels of *NtCAPE* genes under salinity stress. Expression levels were normalized to the internal reference gene (ACTIN) and are presented relative to the corresponding control samples. Data represent the mean ± SD of three biological replicates. The qRT-PCR results show expression trends consistent with the RNA-seq analysis, confirming the reliability of the transcriptomic data. GraphPad prism software 8.3.0 was used for the statistical analysis of this figure; Significant differences were determined by one-way ANOVA followed by the LSD multiple comparison test. ns, not significant; * *p* < 0.5, ** *p* < 0.01, *** *p* < 0.001, **** *p* < 0.0001.

To validate the RNA-seq results, a subset of *NtCAPE* genes showing representative expression patterns was selected for qRT-PCR analysis under water-deficit stress and salinity stress conditions (Figure 7). Consistent with the RNA-seq data, qRT-PCR results confirmed that selected *NtCAPE* genes were significantly induced by water-deficit stress, with expression levels increasing at later time points in *NtCAPE9* and *NtCAPE17* and decreasing at later time points in *NtCAPE2* and *NtCAPE5* compared with control conditions (Figure 7A). Similarly, under salinity stress, the tested genes exhibited expression trends that closely matched the RNA-seq profiles, including both stress-induced and stress-repressed patterns (Figure 7B). Overall, the strong concordance between qRT-PCR and RNA-seq data demonstrates the reliability of the transcriptomic analysis and confirms that *NtCAPE* genes are responsive to water-deficit stress and salinity stress, reinforcing their putative roles in abiotic stress signaling.

### 2.9. NtCAPE9 Alleviates Salinity-Stress-Induced Yellowing and Promotes Seed Germination in Tobacco

Since *NtCAPE9* was upregulated under salinity stress, we hypothesized that the mature NtCAPE9 peptide may participate in the tobacco salinity-stress response. To test this, the mature NtCAPE9 peptide was synthesized and applied exogenously. Detached leaf discs from 6-week-old K326 tobacco plants were treated with NaCl in the presence or absence of NtCAPE9 peptide, and salinity-stress-induced yellowing was evaluated.

**Figure 8:**
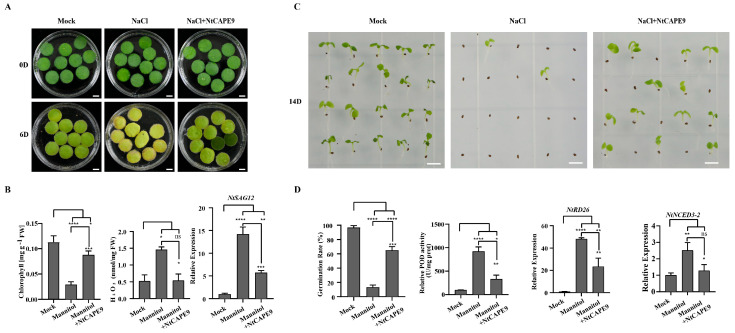
Role of NtCAPE9 peptide in salinity stress responses. (**A**) Phenotypic changes in tobacco detached leaf discs treated with Mock, NaCl, or NaCl + NtCAPE9 peptide at 0 and 6 days. (**B**) Quantification of chlorophyll content, relative expression levels of *NtSAG12* and H_2_O_2_ content in detached leaf discs under the indicated treatments. (**C**) Effect of NtCAPE9 peptide on tobacco seed germination under salinity stress. Representative images were taken after 14 days of treatment. (**D**) Quantification of seed germination rate, relative expression levels of *NtNCED3-2* and *NtRD26*, and relative POD activity under the indicated treatments. Data are presented as the mean ± SD of three independent experiments. Error bars indicate SD. Significant differences in NaCl and NaCl + *NtCAPE9* treatments compared with Mock were determined by one-way ANOVA. The direct comparison between NaCl and NaCl + NtCAPE9 was analyzed using an unpaired Student’s *t*-test. ns, not significant; * *p* < 0.5; ** *p* < 0.01; *** *p* < 0.001; **** *p* < 0.0001. Scale bar = 1 cm.

As shown in Figure 8A, leaf discs from all treatments showed a similar green phenotype at 0 D. After 6 days, salinity stress treatment caused obvious yellowing compared with the Mock control, indicating that salinity stress accelerated chlorophyll degradation and leaf senescence. In contrast, leaf discs treated with NaCl together with NtCAPE9 remained visibly greener than those treated with salinity stress alone, suggesting that NtCAPE9 alleviated salinity-induced senescence symptoms. Consistent with the visual phenotype, chlorophyll content was significantly reduced by salinity stress treatment, whereas exogenous NtCAPE9 significantly improved chlorophyll retention under salinity stress (Figure 8B). To further confirm the effect of NtCAPE9 on salinity-stress-induced senescence, the expression of senescence-related genes was analyzed. Salinity stress treatment strongly induced the senescence marker gene *NtSAG12*, while the addition of NtCAPE9 significantly reduced its expression compared with salinity stress treatment alone (Figure 8B). These results indicate that NtCAPE9 delays salinity-induced senescence and helps maintain photosynthetic activity in tobacco leaf tissues.

Because salinity stress is commonly associated with oxidative damage, H_2_O_2_ accumulation was also measured. Salinity stress treatment increased H_2_O_2_ content compared with the Mock control, whereas NtCAPE9 application reduced H_2_O_2_ accumulation under salinity stress to a level close to the control treatment (Figure 8B). This result suggests that NtCAPE9 may alleviate salinity-stress-associated oxidative damage. The effect of NtCAPE9 on tobacco seed germination under salinity stress was then examined. Under Mock conditions, seeds germinated normally and developed healthy seedlings after 14 days, whereas salinity stress treatment strongly inhibited seed germination and seedling establishment (Figure 8C). In contrast, the addition of NtCAPE9 markedly alleviated the inhibitory effect of salinity stress, resulting in more germinated seeds and better seedling development. Quantitative analysis showed that the germination rate reached 96.67% under Mock conditions but decreased sharply to 13.33% under salinity stress treatment. Exogenous application of NtCAPE9 increased the germination rate to 65.00% under salinity stress (Figure 8D).

To further evaluate salinity-stress-related molecular responses, the expression of *NtNCED3-2* and *NtRD26* was analyzed. Salinity stress treatment significantly induced both genes, whereas NtCAPE9 application reduced their expression levels under salinity stress, especially for *NtRD26* (Figure 8D). In addition, Salinity stress strongly increased POD activity, while NtCAPE9 significantly reduced POD activity compared with the salinity stress treatment alone (Figure 8D). These results are consistent with the reduced H_2_O_2_ accumulation observed in NtCAPE9-treated samples. Together, these findings indicate that exogenous NtCAPE9 peptide alleviates salinity-stress-associated damage under the tested assay conditions, including leaf yellowing, chlorophyll content, senescence-associated gene expression, oxidative stress, stress-responsive gene expression, and seed germination.

### 2.10. Role of NtCAPE17 Peptide in the Osmotic Stress Response of Tobacco

Expression profiling showed that *NtCAPE17* was upregulated under osmotic stress, suggesting that it may participate in tobacco stress adaptation. To further examine this possibility, the mature NtCAPE17 peptide, representing the predicted biologically active processed form of the precursor protein, was chemically synthesized and applied exogenously. Detached leaf discs from 6-week-old K326 tobacco plants were incubated in buffer containing 300 mM mannitol with or without 1 μM NtCAPE17 peptide to evaluate its effect on osmotic-stress-induced leaf yellowing.

**Figure 9:**
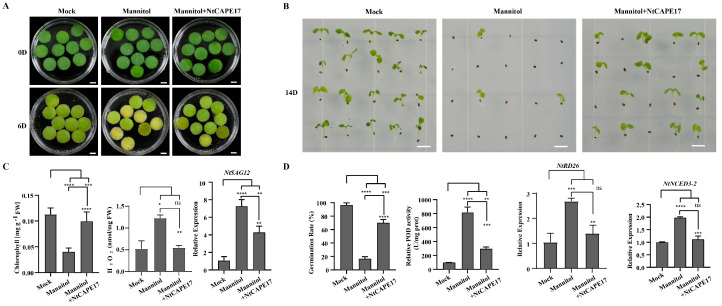
Role of NtCAPE17 peptide in osmotic stress responses. (**A**) Phenotypic changes in tobacco detached leaf discs treated with Mock, mannitol, or mannitol + NtCAPE17 peptide at 0 and 6 days. Detached leaf discs from 6-week-old K326 plants were floated in treatment buffer containing 300 mM mannitol with or without 1 μM NtCAPE17 peptide. (**B**) Quantification of chlorophyll content, relative expression levels of *NtSAG12*, and H_2_O_2_ content in detached leaf discs under the indicated treatments. (**C**) Effect of NtCAPE17 peptide on tobacco seed germination under osmotic stress. Representative images were taken after 14 days of treatment. (**D**) Quantification of seed germination rate, relative expression levels of *NtNCED3-2* and *NtRD26*, and relative POD activity under the indicated treatments. Data are presented as the mean ± SD of three independent experiments. Error bars indicate SD. Significant differences in mannitol and mannitol + NtCAPE17 treatments compared with Mock were determined by one-way ANOVA. The direct comparison between mannitol and mannitol + NtCAPE17 was analyzed using an unpaired Student’s *t*-test. ns, not significant; * *p* < 0.5, ** *p* < 0.01, *** *p* < 0.001, **** *p* < 0.0001. Scale bars = 1 cm.

As shown in Figure 9A, leaf discs from all treatments showed a similar green phenotype at 0 D. After 6 days, mannitol treatment caused obvious yellowing compared with the mock control, indicating that osmotic stress promoted chlorophyll degradation and leaf senescence. In contrast, leaf discs treated with mannitol together with NtCAPE17 remained visibly greener than those treated with mannitol alone, suggesting that exogenous NtCAPE17 alleviated osmotic stress-induced senescence symptoms. Consistent with the visual phenotype, chlorophyll content was significantly reduced under mannitol treatment, whereas NtCAPE17 application markedly improved chlorophyll retention under osmotic stress (Figure 9B). To further evaluate the senescence-related responses, the expression of *NtSAG12* was examined. Osmotic stress strongly induced the senescence positive marker gene *NtSAG12*, while the addition of NtCAPE17 significantly reduced its expression compared with mannitol treatment alone (Figure 9B). These results further support that NtCAPE17 delays osmotic-stress-induced senescence and helps maintain photosynthetic capacity.

Because osmotic stress is commonly associated with oxidative damage, H_2_O_2_ accumulation was also measured. mannitol treatment significantly increased H_2_O_2_ content, whereas NtCAPE17 application reduced H_2_O_2_ accumulation to a level much closer to the mock control (Figure 9B). This indicates that NtCAPE17 may alleviate osmotic-stress-associated oxidative damage. The effect of NtCAPE17 on seed germination under osmotic stress was then analyzed. Under mock conditions, tobacco seeds germinated normally after 14 days, while osmotic stress strongly inhibited germination and seedling establishment (Figure 9C). Notably, the inhibitory effect of osmotic stress was clearly alleviated by exogenous NtCAPE17, resulting in more germinated seeds and better seedling growth. Quantitative analysis showed that the germination rate was approximately 96.67% under mock conditions, decreased sharply to 16.67% under mannitol treatment, and increased to 70.00% when NtCAPE17 was added together with mannitol (Figure 9D).

To further investigate stress-related molecular responses, the expression of *NtNCED3-2* and *NtRD26* was analyzed. mannitol treatment significantly induced NtNCED3-2 expression, whereas NtCAPE17 application reduced its expression to a level comparable to the mock control (Figure 9D). *NtRD26* was also induced by mannitol, and NtCAPE17 treatment showed a decreasing trend, although the difference compared with mannitol alone was not significant (Figure 9D). In addition, osmotic stress strongly increased POD activity, while NtCAPE17 significantly reduced POD activity under osmotic stress (Figure 9D), consistent with the reduced H_2_O_2_ accumulation observed in NtCAPE17-treated samples. Together, these results demonstrate that exogenous NtCAPE17 peptide alleviates osmotic-stress-associated damage under the tested assay conditions.

## 3. Discussion

PR1/CAP proteins are a family of small, secreted proteins with key roles in plant defense and development. Although *PR1/CAP* genes have been identified in several crops [13,19,20,21], CAPE-producing PR1 members remained uncharacterized in tobacco. In this study, we identified 17 CAPE-producing *PR1* genes in the tobacco genome and characterized their conserved domains, motifs, and gene structures, phylogeny, promoter features, and expression profiles. These analyses provide a foundation for understanding the potential roles of *NtCAPE* genes in tobacco stress responses.

All NtCAPE proteins possess negative GRAVY values, indicating hydrophilic properties consistent with PR1 proteins in other species [22], and are predicted to localize to the vacuole [4,23]. The conserved C-terminal CAPE motifs suggest that NtPR1 proteins may serve as precursors of mature bioactive CAPE peptides. In tomato, CAPE1 is derived from PR1b via proteolytic cleavage at the conserved CNYx motif [5]; in Arabidopsis, XCP1 cleaves PR1 at the CNYD motif to release AtCAPE9 in a salicylic acid-promoted manner [9]. However, the protease(s) responsible for NtCAPE peptide maturation in tobacco remain unknown. NtCAPE9 and NtCAPE17 are likely generated from PR1 precursors by unknown XCP1-like or apoplastic proteases, which should be experimentally validated in future studies.

Phylogenetic analysis, conserved motif examination, and gene structure characterization revealed broad structural conservation together with moderate diversification within the NtCAPE family. The grouping of NtCAPE proteins into distinct clades and the similar exon–CDS structures among closely related members indicate conserved structural features within specific subgroups. These patterns are consistent with possible duplication followed by divergence, although the present phylogenetic and gene-structure data cannot fully resolve the evolutionary history or functional consequences of these events. Most *NtCAPE* genes showed compact, CDS-dominated structures, suggesting conservation of the core PR1/CAP-coding region; however, this alone does not prove strong evolutionary constraint or functional equivalence among members [24,25]. This interpretation is consistent with the divergent expression patterns observed among *NtCAPE* genes, but the regulatory significance of these UTR differences remains to be experimentally validated [26]. Chromosomal distribution and synteny analyses suggested that tandem and segmental duplication events contributed to NtCAPE family expansion. Consistently, Ka/Ks analysis of duplicated *NtCAPE* gene pairs showed that all calculable WGD/segmental duplicated pairs had Ka/Ks values below 1, suggesting that these duplicated genes have largely evolved under purifying selection after duplication. In contrast, the tandem duplicated pairs *NtCAPE12/NtCAPE14* and *NtCAPE13/NtCAPE15* showed 0 values of Ka and Ks, indicating nearly identical coding sequences and precluding reliable inference of selection pressure from Ka/Ks ratios for these pairs. Comparative phylogenetic analysis across representative plant species further revealed both conserved grouping patterns and species-biased diversification among CAPE-producing PR1 proteins. These findings support a model in which duplication-driven expansion was followed by constrained sequence diversification within the NtCAPE family [27,28]. Nevertheless, resolving the temporal order of duplication events and the deeper evolutionary origin of individual NtCAPE loci will require broader taxon sampling and more dedicated evolutionary reconstruction.

Expression analyses provided an overview of the stress-associated expression patterns of *NtCAPE* genes under water-deficit stress and salinity stress. Although the public RNA-seq heatmaps were interpreted as descriptive expression profiles rather than formal differential expression evidence, they revealed apparent gene-specific variation within the NtCAPE family. Such divergent expression patterns suggest that NtCAPE members may not function identically; some members may be preferentially associated with specific stress contexts, whereas others may exhibit more stable expression or potential functional redundancy. This view is consistent with observations in other plant species, where *PR1/CAP* family members display both overlapping and distinct expression profiles [6,29]. qRT-PCR validation further supported the stress-associated expression of selected *NtCAPE* genes. The family-wide characterization and stress-associated expression patterns further showed *NtCAPE9* displayed the most prominent salinity-associated expression profile and represents a relatively distinct member of the family, as it belongs to Clade III and is located on Chr13 without obvious syntenic connections. By contrast, *NtCAPE17* showed a pronounced late response under water-deficit stress/osmotic stress-associated conditions and belongs to Clade IV, which includes the expanded Chr23 *NtCAPE* cluster. In addition, promoter analysis revealed that both genes harbor multiple stress- and hormone-responsive cis-acting elements, supporting their potential association with abiotic stress-related regulation. Therefore, *NtCAPE9* and *NtCAPE17* were selected as representative candidates that differ in stress-associated expression, promoter architecture, and evolutionary position within the NtCAPE family.

The biological activities observed for the synthetic mature NtCAPE9 and NtCAPE17 peptides suggest that selected NtCAPE-derived peptides may intersect with major phytohormone and redox-associated stress pathways, particularly ABA-, SA-, and ROS-related signaling. Exogenous NtCAPE9 and NtCAPE17 treatment downregulated *NCED3* (an ABA biosynthesis gene) and *RD26* (an ABA-responsive NAC transcription factor) under salinity and osmotic stress (Figure 8D and Figure 9D), suggesting a potential modulation of ABA-associated stress signaling. Meanwhile, the involvement of SA in CAPE-mediated immunity has been well documented in Arabidopsis and tomato [8,9], raising the possibility that NtCAPE peptides may coordinate crosstalk between the ABA and SA pathways to balance stress adaptation and defense responses. Furthermore, the observed reduction in H_2_O_2_ accumulation and POD activity in peptide-treated samples points to a role for ROS/RNS signaling in *NtCAPE*-mediated stress tolerance, consistent with recent studies highlighting ROS-driven signaling pathways in stress-responsive gene regulation [30,31]. However, because endogenous hormone levels, ROS dynamics, and early signaling events (e.g., MAPK activation) were not directly measured in this study, these mechanistic interpretations remain hypotheses requiring further experimental validation.

After proteolytic release, mature NtCAPE peptides are expected to be perceived at the cell surface; however, their specific receptor(s) remain unknown. Because CAPE peptides are derived from secreted PR1/CAP proteins, NtCAPE9 and NtCAPE17 are likely to act as extracellular signaling peptides perceived by unknown plasma membrane-localized receptor-like kinases or receptor-like proteins. In Arabidopsis, XCP1-mediated cleavage of PR1 releases *AtCAPE9* and activates systemic immunity [9], supporting the importance of proteolytic maturation in CAPE signaling. The stress-protective effects of *NtCAPE9* and *NtCAPE17* observed in this study may therefore involve an unknown receptor-mediated extracellular signaling pathway that activates downstream defense and stress-response networks. Future studies should aim to identify the corresponding processing enzymes and receptor systems in tobacco.

Although exogenous peptide assays demonstrated the biological activity of *NtCAPE9* and *NtCAPE17,* their physiological relevance in vivo should be interpreted with caution. Our results show that these peptides are biologically active under salinity and osmotic stress, particularly during seed germination and detached-leaf stress responses. Evidence from Arabidopsis shows that *AtCAPE1* regulates seedling salinity-stress sensitivity, while PR1-derived *AtCAPE9* contributes to systemic immunity [9,32]. In tomato, CAPE1 restricts bacterial colonization through plant-mediated defense activation rather than direct antibacterial activity [8]. These findings suggest that NtCAPE peptides may function as extracellular signals coordinating whole-plant stress adaptation. However, the whole-plant relevance of *NtCAPE9*- and *NtCAPE17*-mediated stress tolerance remains unknown, and the exogenous peptide bioactivity demonstrated here does not directly prove that endogenous *NtCAPE* genes are naturally processed to produce active CAPE peptides in vivo.

Collectively, our results indicate that at least some NtCAPE members encode predicted CAPE precursors whose mature synthetic peptides display stress-associated activity under the tested assay conditions. Exogenous NtCAPE9 and NtCAPE17 helped maintain leaf greenness and chlorophyll content, suppressed senescence-associated gene expression, reduced H_2_O_2_ accumulation, modulated stress-responsive gene expression, and improved seed germination under adverse conditions (Figure 8 and Figure 9). These findings are consistent with the growing view that small signaling peptides regulate abiotic stress resilience, leaf longevity, and growth–stress balance in plants [33,34,35]. Nevertheless, several important questions remain. First, bulk-tissue transcriptomics may mask cell-type-specific heterogeneity in *NtCAPE* expression and stress responses; emerging single-cell and spatial transcriptomic approaches [36] offer powerful tools to resolve this spatial complexity. Second, genetic validation using CRISPR/Cas9 knockout or overexpression lines is essential to confirm the endogenous roles of *NtCAPE9, NtCAPE17*, and other *NtCAPE* members in whole tobacco plants. Third, future studies should measure endogenous mature peptide accumulation, identify the proteases and receptors involved in *NtCAPE* processing and perception, and monitor early signaling events such as ROS/RNS production, MAPK activation, and hormone dynamics to establish a complete mechanistic framework for *NtCAPE*-mediated stress adaptation.

## 4. Materials and Methods

### 4.1. Plant Growth and Treatment

To examine stress-induced expression patterns, *N. tabacum* cv. K326 was used in this study. Seeds were surface-sterilized with 70% (*v*/*v*) ethanol for 1 min, followed by treatment with 15% (*w*/*v*) H_2_O_2_ for 12 min, and rinsed thoroughly (4–5 times) with sterile distilled water. After germination, seedlings were grown on Murashige and Skoog (MS) medium in a growth chamber (Conviron, Winnipeg, MB, Canada) at 25 °C under long-day conditions (16 h light/8 h dark). Four-week-old seedlings were transferred to fresh MS medium for stress treatments. For water-deficit stress simulation, seedlings were incubated in MS liquid medium supplemented with 20% (*w*/*v*) PEG 6000 and harvested at 0, 1, 2, 4, and 8 h after treatment. For salinity stress, seedlings were treated with 150 mM NaCl and sampled at 0 and 1 h. Whole seedlings were collected, immediately frozen in liquid nitrogen, and stored at −80 °C until further analysis.

### 4.2. Genome-Wide Identification of NtCAPE Genes

To identify *NtCAPE* genes, eight Arabidopsis CAPE protein sequences were retrieved from The Arabidopsis Information Resource (TAIR). Domain analysis using the Pfam database [37] confirmed the presence of the conserved CAP domain (PF00188). The genome assembly and corresponding protein sequences of *N. tabacum* were obtained from the Nicomics database [38]. The hidden Markov model (HMM) profile of the CAP domain was downloaded from Pfam and used as a query to search candidate NtCAPE proteins in the tobacco proteome using HMMER (v3.3.2) with an E-value threshold of 1 × 10^−20^. In parallel, Arabidopsis CAPE protein sequences were used as queries for BLASTP searches against the tobacco protein database. Sequences identified from both approaches were combined, and redundant entries were removed. All candidate proteins were further validated for the presence of the CAP domain using the Conserved Domain Database (CDD) and SMART [39]. Proteins lacking the conserved CAP domain were excluded from subsequent analyses. To further ensure that only bona fide CAPE-producing PR1 genes were retained, MEME motif analysis (v5.5.0) was performed on all CAP domain-containing candidates to identify the conserved C-terminal CAPE peptide region containing the characteristic PPGNxxxxPY signature. Only candidates satisfying both criteria—presence of the conserved CAP domain and the C-terminal CAPE signature motif—were designated as *NtCAPE* genes. The non-redundant NtCAPE proteins containing a complete CAP domain were subjected to further bioinformatic analyses, including phylogenetic analysis, chromosomal localization, duplication pattern analysis, cis-regulatory element prediction, and expression profiling.

### 4.3. Physicochemical Properties, Phylogenetic Relationships, Gene Structure, and Conserved Motifs Analysis

The isoelectric point (pI), amino acid length, and molecular weight of NtCAPE proteins were calculated using the ProtParam tool in ExPASy [40]. Signal peptides were predicted using SignalP v6.0 with default parameters, and subcellular localization was predicted using the WoLF PSORT program) (https://wolfpsort.hgc.jp/ accessed on 21 April 2026) [41]. CAPE protein sequences from *Arabidopsis* (*AtCAPE*), tomato (*SlCAPE*), pepper (*CaCAPE*), potato (*StCAPE*), alfalfa (*MtCAPE*), eggplant (*SmCAPE*), and tobacco (*NtCAPE*) were used for phylogenetic analysis. Phylogenetic trees were constructed using the maximum-likelihood method in MEGA 11 based on full-length protein sequences. The best-fitting amino acid substitution model was selected according to the model test implemented in MEGA 11. Branch support was evaluated using 1000 bootstrap replicates (Supplementary Table S3) [42]. The resulting trees were visualized with branch lengths and bootstrap values. Trees were treated as unrooted, and evolutionary interpretations were restricted to sequence clustering and relative relationships among the analyzed proteins. Gene structure analysis was conducted by comparing genomic DNA sequences with corresponding coding sequences (CDS), and exon-intron structures were visualized using GSDS 2.0 [43]. Conserved motifs of NtCAPE proteins were identified using MEME v5.5.9 with the maximum number of motifs set to 10 and other parameters at default settings [44]. Pairwise sequence similarity analysis was performed using full-length amino acid sequences, and sequence identity values were calculated using TBtools-Il (Toolbox for Biologists) v2.467 [45]. The similarity matrix was visualized as a heatmap.

### 4.4. Chromosomal Locations and Gene Duplications Analysis

The genomic positions of *NtCAPE* genes were obtained from the *N. tabacum* genome GFF3 annotation file and visualized on corresponding chromosomes using TBtools [45]. Intra-genomic collinearity analysis was performed using the MCScanX program integrated in TBtools. Protein sequences of *NtCAPE* genes were subjected to all-against-all BLASTP searches with an E-value threshold of 1 × 10^−5^ to identify potential homologous gene pairs. Segmental or WGD-derived gene pairs were identified from MCScanX collinearity results based on collinear blocks containing at least five syntenic gene pairs and ≥50% sequence identity. Tandem duplication was defined as two or more *NtCAPE* genes located within a 200 kb genomic interval on the same chromosome, with sequence identity ≥70% at the protein level. For duplicated gene pairs, Ka/Ks ratios were calculated using Ka/Ks_Calculator 2.0. The duplicated gene pairs, duplication type, Ka, Ks, and Ka/Ks values are listed in Supplementary Table S1.

### 4.5. Prediction and Classification of Cis-Regulatory Elements

The 2000 bp upstream sequences from the transcription start site (TSS) of each *NtCAPE* gene were extracted from the tobacco genome. Cis-regulatory elements (CAREs) within these promoter regions were identified using the PlantCARE database [46,47]. Based on functional annotations provided by PlantCARE, identified elements were grouped into three categories: growth and development-related elements, phytohormone-responsive elements, and stress-responsive elements. The frequency of each cis-element type was calculated and visualized as a heatmap using TBtools.

### 4.6. Expression Analysis of NtCAPE Genes Under Abiotic Stress

To examine *NtCAPE* gene expression under abiotic stress conditions, public RNA-seq datasets of *N. tabacum* cv. K326 were downloaded from the NCBI Sequence Read Archive (SRA), including PRJNA883680 for water-deficit stress and PRJNA532660 for salinity stress. Detailed sample information, including SRA run accessions, BioSample IDs, treatment conditions, sampling time points, and biological replicate information, is provided in Supplementary Table S4. Raw reads were quality-checked using FastQC v0.11.9 and processed with Trimmomatic v. 0.39 to remove adaptor sequences, low-quality reads, and low-quality bases. Clean reads were aligned to the *N. tabacum* reference genome using HISAT2 v2.1.0 after construction of the genome index. Gene-level expression was quantified from the resulting BAM files using StringTie v2.2.1 with the corresponding genome annotation file. Expression abundance was calculated as transcripts per million (TPM). TPM values of *NtCAPE* genes were extracted from the expression matrix and transformed as log_2_ (TPM + 1) for heatmap visualization. Heatmaps were generated using TBtools.

### 4.7. Validation of RNA-Seq Data by qRT-PCR Analysis

To validate the RNA-seq results under water-deficit stress and salinity stress conditions, representative *NtCAPE* genes showing distinct expression patterns were selected for qRT-PCR analysis. Total RNA was extracted from water-deficit stress- and salinity-treated *N. tabacum* cv. K326 seedlings using a standard RNA extraction protocol. First-strand cDNA was synthesized using PrimeScript RT Master Mix (Takara, Naha, Japan) according to the manufacturer’s instructions. Quantitative real-time PCR (qRT-PCR) was performed using gene-specific primers (Supplementary Table S5) and SYBR Green Master Mix on a real-time PCR detection system. *NtActin* was used as the internal reference gene. Relative expression levels were calculated using the 2^−ΔΔCt^ method [48].

### 4.8. Functional Characterization of NtCAPE Peptides Under Salinity and Osmotic Stress Conditions

Synthetic mature NtCAPE peptides were obtained from GenScript Biologicals (Nanjing, China) with a purity higher than 90%. The peptide sequences were NtCAPE9 (PPGNYIGEKPY) and NtCAPE17 (PPGNFVGQSPY). Stock solutions were prepared in sterilized ddH2O and diluted to the desired working concentration before application.

For stress-induced leaf yellowing assays, detached leaf discs were collected from the middle leaves of 6-week-old tobacco (*N. tabacum* cv. K326) plants grown in the greenhouse. The leaf discs were floated in solutions containing ddH2O, 150 mM NaCl, or 300 mM mannitol. To assess peptide function under stress conditions, 1 μM NtCAPE9 or NtCAPE17 peptide was added to the corresponding treatment solution. NtCAPE9 was applied in the salinity-stress assay, while NtCAPE17 was applied in the osmotic-stress assay. Phenotypic changes were photographed at 0 and 6 days after treatment, and chlorophyll content was subsequently determined [49].

For germination assays, tobacco seeds were grown under control and stress conditions with or without exogenous peptide treatment, following the general approach used for stress-related seed germination assays [50]. Seeds subjected to salinity stress were treated with NaCl in the presence or absence of 1 μM NtCAPE9 peptide, whereas seeds subjected to osmotic stress were treated with 300 mM mannitol in the presence or absence of 1 μM NtCAPE17 peptide. Representative images were taken after 14 days, and germination percentage was calculated for each treatment.

To evaluate oxidative stress responses after peptide treatment, H_2_O_2_ content and peroxidase (POD) activity were measured in detached leaf discs after 6 days of treatment. Fresh samples were immediately frozen in liquid nitrogen and ground into fine powder. H_2_O_2_ content was determined using a hydrogen peroxide assay kit according to the manufacturer’s instructions and expressed as nmol mg^−1^ fresh weight. POD activity was measured using a POD activity assay kit according to the manufacturer’s protocol and expressed as U mg^−1^ protein. The measurement of H_2_O_2_ accumulation and antioxidant enzyme activity followed the general method described by previous studies [50], in which H_2_O_2_ content and POD activities were determined using commercial detection kits to evaluate ROS-related stress responses in tobacco. For gene-expression analysis, samples from the indicated treatments were collected after 6 days, immediately frozen in liquid nitrogen, and used for qRT-PCR analysis of *NtSAG12*, *NtRD26*, and *NtNCED3-2* [48]. Relative expression levels were calculated using the 2^−ΔΔCt^ method. All experiments were repeated three times independently, and each treatment included three biological replicates.

## 5. Conclusions

CAPE-producing *PR1* genes are increasingly recognized as important components of plant defense, development, and stress-adaptation pathways. In this study, 17 CAPE-producing *PR1* genes were systematically identified and characterized in *N. tabacum*. Comprehensive analyses of physicochemical properties, conserved domains, gene structures, chromosomal distribution, duplication patterns, phylogenetic relationships, promoter cis-elements, and predicted functional associations revealed that the *NtCAPE* family is both evolutionarily conserved and functionally diversified.

Transcriptome profiling and qRT-PCR validation further showed that several *NtCAPE* genes respond dynamically to water-deficit stress and salinity stress, suggesting that these genes may participate in abiotic stress-regulatory networks in tobacco. Among them, *NtCAPE9* and *NtCAPE17* were selected for functional validation based on their stress-responsive expression patterns. Exogenous application of the synthetic mature NtCAPE9 peptide alleviated salinity stress-induced leaf yellowing, improved chlorophyll retention, reduced *NtSAG12* expression, decreased H_2_O_2_ accumulation and POD activity, modulated the expression of stress-responsive genes such as *NtNCED3-2* and *NtRD26*, and promoted seed germination under salinity stress. Similarly, NtCAPE17 peptide improved tobacco tolerance to osmotic stress by reducing mannitol-induced yellowing, maintaining chlorophyll content, suppressing senescence-associated responses, reducing oxidative damage, and enhancing seed germination.

Together, these findings provide a genome-wide foundation for understanding CAPE-producing *PR1* genes in tobacco and identify NtCAPE9 and NtCAPE17 as candidate peptide regulators associated with salinity and osmotic stress responses. However, the current functional evidence is mainly based on exogenous peptide assays using detached leaf discs and seed germination systems. Therefore, future studies should focus on detecting endogenous mature CAPE peptides, identifying the proteases and receptors involved in NtCAPE processing and perception, and generating loss-of-function or overexpression lines to confirm the in vivo roles of *NtCAPE* genes in whole-plant stress adaptation.

## Figures and Tables

**Table 1:** Detailed information on CAPE peptides containing *PR1* genes in tobacco.

Gene Name	Sequence ID	C-Terminal CAPE Sequences	Number of AA	Molecular Weight	Theoretical pI	Instability Index	Aliphatic Index	Grand Average of Hydropathicity	Predicted Subcellular Localization
*NtCAPE*1	Nta01g05380.1	PPGNVVGQRPY	163	17,840.89	6.86	16.66	70.67	−0.116	Vacuole.
*NtCAPE*2	Nta01g05390.1	PPGNWRGQRPY	179	19,963.29	8.48	44.52	57.32	−0.488	Vacuole.
*NtCAPE*3	Nta01g05410.1	PPGNYIGERPY	161	17,887.38	6.5	23.31	77.52	−0.177	Vacuole.
*NtCAPE*4	Nta02g21320.1	PPGNYVGQRPY	163	17,973.97	7.58	22.18	68.9	−0.213	Vacuole.
*NtCAPE*5	Nta02g21330.1	PPGNWRGQRPY	179	20,112.45	7.62	37.11	60.56	−0.516	Vacuole.
*NtCAPE*6	Nta02g21350.1	PPGNYIGERPY	161	17,906.36	6.5	24.24	73.91	−0.216	Vacuole.
*NtCAPE*7	Nta03g09700.1	PPGNVIGEKPY	182	19,717.54	8.74	35.12	71.37	−0.172	Vacuole.
*NtCAPE*8	Nta04g09480.1	PPGNVIGEKPY	181	19,665.51	8.92	37.33	70.72	−0.143	Vacuole.
*NtCAPE*9	Nta13g23100.1	PPGNYIGEKPY	201	23,316.64	7.55	38.08	72.79	−0.327	Vacuole.
*NtCAPE*10	Nta20g10300.1	PPGNVIGQSPY	168	18,499.51	5.26	29.31	70.77	−0.223	Vacuole.
*NtCAPE*11	Nta23g22890.1	PPGNYRGESPY	168	18,587.53	4.83	24.34	71.37	−0.303	Vacuole.
*NtCAPE*12	Nta23g22900.1	PPGNYRGESPY	168	18,573.5	4.83	26.79	71.37	−0.304	Vacuole.
*NtCAPE*13	Nta23g22910.1	PPGNYRGESPY	168	18,586.55	4.96	25.24	71.37	−0.303	Vacuole.
*NtCAPE*14	Nta23g22920.1	PPGNYRGESPY	168	18,573.5	4.83	26.79	71.37	−0.304	Vacuole.
*NtCAPE*15	Nta23g22930.1	PPGNYRGESPY	168	18,586.55	4.96	25.24	71.37	−0.303	Vacuole.
*NtCAPE*16	Nta23g22940.1	PPGNYRGESPY	168	18,573.5	4.83	26.79	71.37	−0.304	Vacuole.
*NtCAPE*17	Nta23g23130.1	PPGNFVGQSPY	184	20,411.85	5.34	38.35	72.07	−0.16	Vacuole.

## Data Availability

The original contributions presented in this study are included in the article/Supplementary Material. Further inquiries can be directed to the corresponding authors.

## Supplementary Materials

The following supporting information can be downloaded at: https://www.mdpi.com/article/10.3390/plants15121801/s1, Table S1: Sequences of CAPE-producing PR1 proteins and their putative CAPE peptides from representative plant species; Table S2: Ka/Ks analysis of duplicated NtCAPE gene pairs in tobacco; Table S3: Syntenic and homologous relationships between NtCAPE genes and CAPE-related genes from representative plant species; Table S4: Sample information for public RNA-seq datasets used in NtCAPE expression profiling; Table S5: Primer sequences used for qRT-PCR analysis.
